# Impact of therapy escalation on ambulatory care costs among patients with type 2 diabetes in France

**DOI:** 10.1186/1472-6823-13-15

**Published:** 2013-04-29

**Authors:** Florent Guelfucci, Emilie Clay, Samuel Aballéa, Régis Lassalle, Nicholas Moore, Mondher Toumi

**Affiliations:** 1 EPHE Sorbonne, Systèmes intégrés, environnement et biodiversité, (SIEB), Paris, France; 2 University of the Mediterranean Marseille, laboratoire de santé publique, Evaluation des systèmes de soins et santé perçue, Marseille, France; 3 University of Lyon 1, decision sciences and health policies, Lyon, France; 4 INSERM CIC-P 0005 Pharmaco-épidémiologie, Université de Bordeaux 2-CHU Bordeaux, Bordeaux, France

**Keywords:** Cost analysis, Type 2 diabetes mellitus, Oral antidiabetics, Insulin, Renal function

## Abstract

**Background:**

This study compares annual ambulatory care expenditures per patient with type 2 diabetes mellitus (T2DM) in France according to treatment phase and renal function status.

**Methods:**

Records from patients with T2DM were extracted from a health insurance database. Patients were classified in subgroups, by treatment phase: oral/GLP1 monotherapy, double therapy, triple therapy or insulin therapy, and according to renal function status (identified using pharmacy, lab and consultation claims). Annual ambulatory expenditures were estimated from the national insurance perspective by year (from 2005 to 2010) and subgroup.

**Results:**

The number of patients ranged from 9,682 to 11,772 between 2005 and 2010. The average annual expenditure per individual in 2010 ranged from €3,017 (standard deviation: €3,829) for monotherapy to €3,609 ± €3,801 for triple therapy, and €7,398 ± €5,487 with insulin (adjusted ratio insulin therapy/monotherapy: 2.36, p < 0.001). Similar differences between treatement stages were found in previous years. Additional costs for insulin were mainly related to nursing care (multiplied by 18.42, p < 0.001), medical devices and pharmacy costs. DM-attributable drug costs were mainly related to antidiabetic drugs (28% for monotherapy to 71% for triple therapy), but also to cardiovascular system drugs (21% for monotherapy to 51% with insulin) and nervous system drugs (up to 8% with insulin). Declining renal function was associated with an increase in expenses by 12% to 53% according to treatment stage.

**Conclusions:**

Overall, ambulatory care expenditures increase with treatment escalation and declining renal function amongst patients with T2DM. Insulin therapy is associated with substantially increased costs, related to pharmacy, nursing care and medical device costs.

## Background

With the increasing prevalence of treated type 2 diabetes mellitus (T2DM) in France, from 2.6% to 4.4% among people covered by the national health insurance scheme between 2000 and 2009 [1], costs related to diabetes increased from 7.1 billion € in 2001 to 12.5 billion in 2007 [2]. These expenditures represented approximately 8.5% of the National Objective of Healthcare Expenditure (144.8 billion € in 2007).

As an ever-increasing portion of gross domestic product is spent on health care, economic considerations are given increasing importance in the development of treatment guidelines and drug reimbursement decisions. This is illustrated in France by the recent introduction of health economic guidelines by the Haute Autorité de Santé (HAS). The HAS published recommendations for the management of T2DM in France in 2006 [3]. Therapeutic escalation was recommended to maintain glycemic control with an HbA1c < 48 mmol/mol (6.5%) from monotherapy with an oral antidiabetic (Metformine), to oral double therapy, then to triple therapy or insulin therapy. Since the publication of these recommendations, incretin mimetics, i.e. Dipeptidyl peptidase-4 (DPP4) inhibitors and Glucagon-like peptide-1 (GLP-1), have been introduced on the market.

Latest data on the cost of diabetes in France come from the 2007 ENTRED study (Echantillon national témoin représentatif des personnes diabétiques) [4], which described healthcare expenditures in a nationally representative sample of people covered by the largest national health insurance fund, living in metropolitan France. According to this study, average annual individual expenditures for treating people with diabetes were €5,341 in 2007. The first category of expenditure was hospitalisation (over 38%). The second category was drug costs, estimated at €1,343 to €1,402 (25-26%) according to data sources, or €3.67/day to €3.84/day. Antidiabetics represented 23% of the drug costs (oral medications: 13% and insulin: 10%). The main other drug categories were cardiovascular treatments (37% of drug reimbursements), lipid-lowering drugs (9%) and anti thrombotic drugs (6%).

Among T2DM complications, chronic renal disease (CRD) limits the therapeutic options because a reduced glomerular filtration rate results in the accumulation of certain drugs and/or their metabolites. Health care costs among persons with T2DM with end-stage renal failure were estimated to be 3 to 4 times higher compared to T2DM cases without related complications [5].

Several important changes occurred since the ENTRED study. First, new therapeutic classes have been introduced, as previously mentioned. This could have important economic consequences as prices of these new drugs are substantially higher than those of older andiabetics. Secondly, diabetes treatments were intensified in the last decade. According to ENTRED, monotherapy was less frequent in 2007 compared to 2001 (36% vs 41%) while combination of oral antidiabetics (OAD) and combination therapy with insulin were more frequent (34% vs 32% and 21% vs 19%). This trend was pursued in recent years, with use of basal insulins growing at a faster rate than oral antidiabetics [6]. On the other hand, a limitation of the ENTRED study is that it did not provide costs estimations according to treatment escalation.

The objectives of this new study were (1) to update and enhance 2007 ENTRED study figures with data obtained in recent years up to 2010 including the period of launch of new antidiabetic oral treatment (DPP4 inhibitors and GLP1 analogs); (2) to estimate the diabetes-attributable annual expenditures based on a comparison vs. matched individuals without diabetes; (3) to compare annual expenditures per patient between treatment stages and according to renal function status (RFS) and to identify the main cost components driving differences between stages and according to RFS.

## Methods

### Study design and data source

We performed a retrospective cross-sectional analysis using data from the ‘Echantillon Generaliste de Beneficiaires’ (EGB) database. The *EGB database* contains records of reimbursements made by the national health insurance for ambulatory care, for a sample of 530,000 subjects, representative of the general population affiliated with the French health insurance system. The sample is obtained by 1/97th random sampling with control for distribution of age and gender. This anonymized database includes patient demographics, health plan enrolment information, and reimbursed amounts for outpatient procedures, laboratory tests, physician visits and outpatient drug dispensing claims, along with specialty of the prescriber. It contains neither direct clinical information nor results of the lab tests.

EGB is a completely anonymized database with no possible interaction with the persons contributing information. The identification of persons included in the EGB sample is protected by an anonymization process with two cryptographic levels, a procedure conforming to French data protection legislation. The use of EGB for medical research has been approved by the National Commission for Informatics and Liberties (CNIL). The conditions of use of EGB for research purposes and the authorized accesses are stated by law. Researchers involved in this study were authorized to use the database, and have been trained in its use. The study were declared to and approved by the National Institute of Medical Research and Statistics (INSERM).

### Observation period

Patients were monitored between 2005 and 2010 from the date of the first prescription of antidiabetic drug (oral and/or insulin), or from the date of notification of chronic disease/ALD status for T2DM. DM is one of a list of long-term conditions, designated as “Affections Longue Durée” for which patients are entitled to 100% reimbursement of health care. The observation period was partitioned in calendar years. Annual healthcare expenditures in different years were compared between phases of treatment (from 2005 to 2010).

### Patient selection and classification

Patients were included in the treated T2DM cohort if the antidiabetics (ADs) or insulin were dispensed for more than 80% of days over 15-months during the follow-up period. Patients with any evidence of pregnancy, gestational diabetes or any other type of diabetes during the treatment period were excluded.

In absence of complete diagnostic information in the database, patients with declining renal function (DRF) were identified as patients with at least one of the following criteria: (1) at least 2 measurements of creatinine clearance and 2 measurements of urinary protein in one year; (2) at least one long-term condition record with an ICD-10 code related to chronic renal failure; (3) At least one nephrologist consultation associated with a prescription of treatment from the following list: combination of angiotensin converting enzyme inhibitors (ACE-I) and angiotensin II receptor antagonists (A2RA); phosphorus chelators (calcium carbonate, sevelamer, lanthanum, aluminium salts); polystyrene sulfonate); (4) At least two dispensings with an interval from 30 to 365 days of a combination of following treatment: ACE-I, A2RA, phosphorus chelators. This definition was deliberately restrictive in order to minimize the number of false positives.

Based on expert opinion, a patient was considered in a stable treatment stage if he was in that stage for at least 6 months. As pharmacy claims generally occurred at 1-month intervals, patients were classified into oral/GLP1 monotherapy (MT), double therapy (DT), triple therapy (TT) or insulin therapy (IT) (either associated or not with other antidiabetics) if they had at least 6 consecutive pharmacy claims for either one, two, three OAD/GLP-1 analog or insulin (± OAD/GLP-1 analog) treatment without discontinuation. The definition of antidiabetic treatment discontinuation was a gap between two claims of antidiabetic drugs exceeding 6 months. The start date of a treatment phase was the date of the first of the six consecutive claims. The end date of a treatment phase was defined as the date of the first occurring event among the following: (1) a new treatment stage starts; (2) first of 5 consecutive dispensings that do not correspond to the treatment phase; (3) discontinuation.

For each patient, only the years during which the patient was constantly in the same treatment phase were considered. Each year, patients were excluded if they were lost to follow-up (no healthcare resource use during 3 months during the calendar year) or discontinued T2DM treatment.

The estimation of T2DM-attributable expenditures was based on the difference in expenditures between patients with T2DM and matched controls without diabetes. Controls were randomly selected among insured persons included into EGB database not selected above and matched with patients with T2DM according to age and gender. Each year, controls were excluded from the analysis if they had no reimbursement claim during the year or at least one long-term condition record with an ICD 10 code related to chronic kidney failure before or during the year of interest.

### Estimation of yearly reimbursed expenditures

Total amounts charged by providers and amounts reimbursed by the national health insurance are included in all medical claim records. Therefore healthcare expenditures from the health insurance perspective were calculated by summing amounts reimbursed by national health insurance for all healthcare resources consumed by calendar year, overall and by type of resource. All ambulatory care resources were included: pharmacy, physician visits and consultations, nursing care, diagnostic tests, imaging procedures, physiotherapy, medical devices, transportation… Two types of expenditures were reported: total expenditures (sum of all reimbursed amounts, related to diabetes or not) and expenditures attributable to T2DM, estimated as the mean differences in reimbursed expenditures between patients with T2DM and matched controls without T2DM.

### Statistical analysis

In order to estimate expenditures attributable to diabetes, each case was matched to 2 controls without diabetes with same year of birth and same gender, using a greedy matching algorithm. In addition, to estimate the effect of treatment phase, patients with T2DM in a given treatment phase were matched to patients with T2DM at earlier treatment phase in same calendar year, with same year of birth and gender. The matching ratio was 1:2 for DT vs MT; 1:3 for IT vs DT; and 1:2 for IT vs MT. The matching algorithms were applied for each year successively.

Effects of treatment stages on annual ambulatory care expenditures were analysed by means of generalised linear models with a log link function and negative binomial distribution, adjusting on patient characteristics not included in matching variables (area of residence, eligibility for full reimbursement of healthcare for people who cannot afford a private top-up health insurance), calendar year, and clinical characteristics such as co-prescriptions (cardiovascular and antihypertensive drugs) and long terms conditions. All analyses were performed using SAS software version 9.1.

## Results

**Figure 1:**
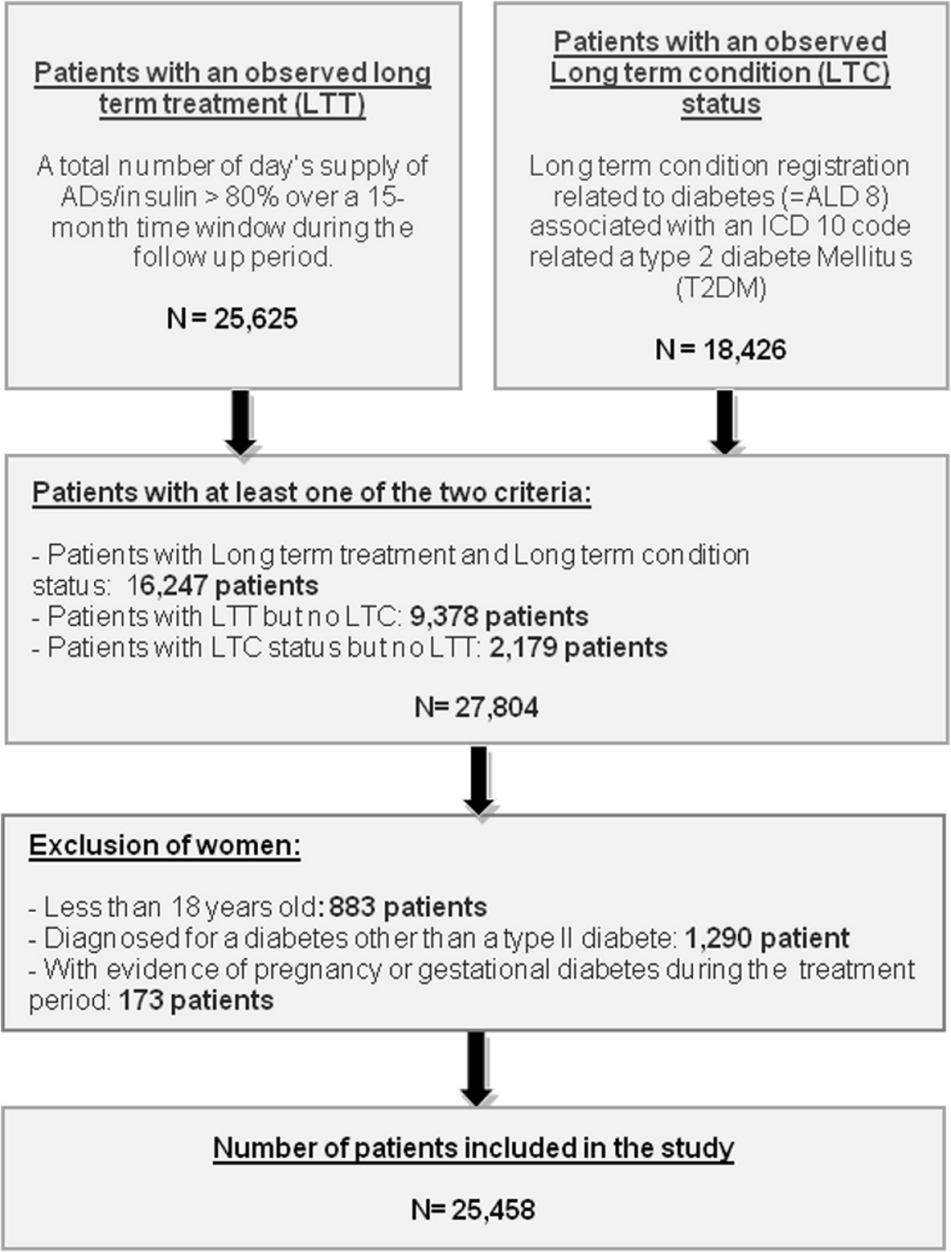
Selection of patient with T2DM.

A total of 25,458 patients treated for T2DM were selected. The proportion of patients treated was stable over time (Figure 1; Table 1). 2,535 (10%) patients had DRF (Table 2). The number of patients continuously treated in one phase over a calendar year ranged from 9,682 to 11,772 between 2005 and 2010. The proportion of patients in IT among patients continuously treated in one phase, increased from 10.7% in 2005 to 13.9% in 2009 (Table 1). Each year, around half of the patients were excluded from the transversal analysis due to the high proportions of patients lost to follow-up or discontinuing their treatment.

**Table 1:** Number of patients analyzed

**Year**	**2005**		**2006**		**2007**		**2008**		**2009**		**2010**	
**Diabetics patients followed up (1)**	**19,005**		**20,434**		**21,512**		**22,566**		**23,713**		**24,862**	
**Patients analyzed**	**9,682**		**10,397**		**11,094**		**11,772**		**11,583**		**9,734**	
Monotherapy	5,792	59.8%	5,890	56.7%	6,080	54.8%	6,390	54.3%	6,065	52.4%	4,764	
Double therapy	2,484	25.7%	2,815	27.1%	3,029	27.3%	3,147	26.7%	3,100	26.8%	2,770	28.5%
Triple therapy	355	3.7%	447	4.3%	551	5.0%	657	5.6%	730	6.3%	803	8.2%
Polytherapy	14	0.1%	28	0.3%	44	0.4%	66	0.6%	73	0.6%	98	1.0%
Insulinotherapy	1,037	10.7%	1,217	11.7%	1,390	12.5%	1,512	12.8%	1,615	13.9%	1,299	13.3%
**Patients excluded**	**9,323**		**10,037**		**10,418**		**10,794**		**12,130**		**15,128**	
Lost to follow up	4,562	49%	4,877	49%	4,909	47%	4,821	45%	4,923	41%	4,418	29%
No pharmacological treatment	839	9%	1,152	11%	1,466	14%	1,719	16%	2,008	17%	1,610	11%
**Pharmacological treatment with:**												
Discontinuation period	1,692	18%	1,846	18%	1,907	18%	1,959	18%	2,535	21%	6,411	42%
Non stationary period	1,255	13%	1,070	11%	1,086	10%	1,225	11%	1,432	12%	1,978	13%
Stage changes	975	10%	1,092	11%	1,050	10%	1,070	10%	1,232	10%	711	5%

**Table 2:** Definition of diabetic patients with declining renal function

**Diabetic patients**		**25,458**	
	2 Measurements of creatinine clearance and 2 Measurements of urinary protein within a one-year time frame	2,109	8,3%
OR	At least one record associated with a long term condition “chronic nephropathy and primitive nephrotic syndrom” and at least one ICD 10 code related to chronic kidney failure	127	0,5%
OR	At least one prescription dispensing of treatment from the following list associated to a nephrologist visit: ACE-I (Angiotensin converting enzyme inhibitors) and A2RA (Angiotensin II receptor antagonists) in association, phosphorus chelators (*), polystyrene sulfonate (**), Angiotensin converting enzyme (ACE) inhibitors,	496	1,9%
OR	At least two dispensings with an interval from 30 to 365 days of a conbination of following treatment: ACE-I, A2RA, phosphorus chelators (*), polystyrene sulfonate (**)	170	0,7%
**Total number of patients with a chronic kidney failure (CKF) (Declining Renal Function)**		**2,535**	**10,0%**
**Patients with terminal CKF: patients with a CKF and…:**			
	At least 2 records associated with a CCAM code related to dialyse within a One-week time period	67	0,3%
OR	At least 2 prescription dispensings of erythropoietin AND 2 prescriptions dispensing of phosphorus chelators within a 45 days-time period	22	0,1%
**Total number of patients with a terminal CKF**		**80**	0,3%
**Patients with severe CKF: patients with a CKF…**		**585**	2,3%
	At least 2 prescription dispensings of erythropoietin	470	1,8%
OR	At least 2 prescription dispensings of phosphorus chelators	140	0,5%
OR	At least 4 nephrologist visits within 12-months time frame	58	0,2%
**Total number of patients with a severe CKF**		**585**	2,3%
**Patients with moderate CKF: Patients with a CKF not defined as severe or terminal**			
	**Total number of patients with moderate CKF**	**1,870**	7,3%

The average age was between 64 and 68 years old in 2010 among patients with normal renal function (NRF), according to treatment phase, increasing by 1 to 2 years compared to 2005. The prevalence of specific cardiovascular drug co-prescriptions was higher in patients with T2DM than in the control group, and was the highest for those treated by insulin. All patients with diabetes are entitled to full reimbursement health care expenses related to diabetes and associated long-term conditions, therefore average reimbursement rates of antidiabetics are high, from 90% to 98% (Table 3). There was no major difference in demographic characteristics between patients with or without DRF.

**Table 3:** Socio-demographic and clinical characteristics by treatment stage and renal function status among patients treated for T2DM in 2010

	**Patients with normal renal function**					**Patients with declining renal function**			
	**Mono-therapy**	**Double therapy**	**Triple therapy**	**Insulin therapy**	**Non diabetic patients**	**Mono-therapy**	**Double therapy**	**Triple therapy**	**Insulin therapy**
Number of patients	4,353	2,503	728	1,032	27,389	411	267	78	267
Female (N,%)	48.0%	46.1%	43.4%	53.7%	47.6%	45.3%	44.9%	35.9%	47.2%
Age (mean, sd) (1)	67.7 (11.5)	66.3 (11.1)	64.0 (10.1)	67.4 (11.9)	66.9(11.4)	69.6 (11.0)	67.3 (11.6)	66.0 (10.3)	71.4 (10.0)
Out of metropolitan France	4.5%	5.3%	5.9%	8.6%	-	6.6%	9.4%	7.7%	10.1%
CMU* beneficiary	2.5%	3.4%	4.3%	3.3%	3.3%	4.9%	6.0%	3.9%	3.0%
Reimbursement rate for antidiabetics (mean, sd)	90.9 (15.6)	95.7 (12.0)	97.2 (10.3)	98.1 (8.1)	-	-	-	-	-
Antihypertensive	79.0%	79.0%	81.2%	86,0%	47.8%	88.3%	85.8%	93.6%	96.3%
Antithrombotic agents	41.3%	41.9%	40.3%	60.3%	22.7%	49.4%	44.9%	32.1%	74.5%
Hypolipidemic drugs	62.8%	65.0%	72.1%	69.0%	33.0%	67.4%	67.8%	71.8%	79.8%
ALD*-5: Cardiac Insufficiency	2.8%	1.8%	1.7%	2.4%	1.7%	4.4%	2.3%	2.6%	1.9%
ALD-8: Diabetes	32.9%	50.1%	62.9%	75.6%	-	41.6%	56.6%	73.1%	77.9%
ALD-12: Hypertension	10.9%	9.8%	9.9%	10.2%	3.0%	13.1%	18.0%	14.1%	12.0%
ALD-13: Myocardial infarction	5.7%	5.1%	3.7%	5.6%	3.7%	7.5%	3.8%	1.3%	7.1%
ALD-23: Psychosis	4.0%	4.0%	5.5%	6.5%	3.5%	2.9%	3.8%	2.6%	4.5%
ALD-30: Malignant cancer	5.8%	5.0%	3.2%	3.5%	5.5%	7.1%	6.7%	2.6%	5.6%

*CMU = Couverture maladie universelle (Universal health care coverage).

### Ambulatory care expenditures over time

**Figure 2:**
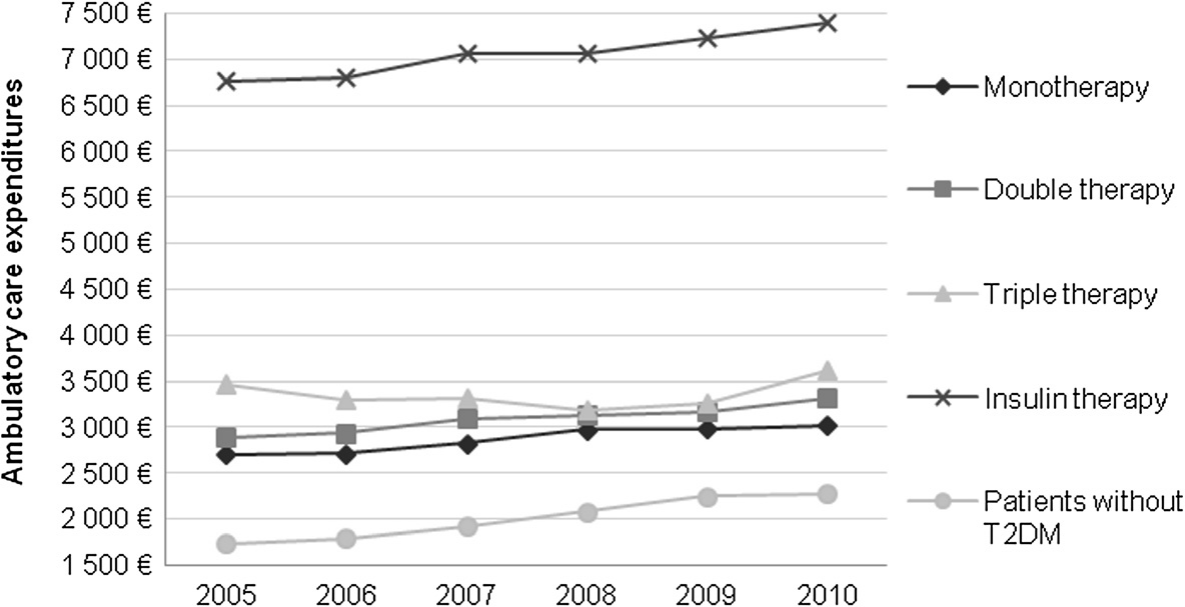
Total ambulatory care individual expenditures (€) according to time and treatment stage.

Individual annual ambulatory care expenditures increased from €2,703 in 2005 to €3,017 in 2010 for patients in MT (+11.6%), from €2,884 to €3,308 in DT (+14.7%) and from €6,759 to €7,398 in IT (+9.4%) (Figure 2; Table 4). These increase rates are lower than the rate for controls without diabetes (+26.3%). Quasi-stability is observed for patients in TT (+0.2% from €3,473 to €3,482) (Figure 2).

**Table 4:** Total ambulatory care expenditures according to time and treatment phase for patients (€)

	**2005**			**2006**			**2007**			**2008**			**2009**			**2010**		
	**N**	**Mean**	**SD**	**N**	**Mean**	**SD**	**N**	**Mean**	**SD**	**N**	**Mean**	**SD**	**N**	**Mean**	**SD**	**N**	**Mean**	**SD**
**Patients with Normal Renal Function**																		
**Monotherapy**	**5,507**	2,703	3,333	5,543	2,711	3,407	5,682	2,821	3,542	5,921	2,971	3,916	5,538	2,980	3,876	4,353	3,017	3,829
**Double therapy**	2,324	2,884	3,113	2,587	2,935	3,206	2,762	3,095	3,622	2,839	3,125	3,611	2,786	3,171	3,861	2,503	3,308	4,045
**Triple therapy**	328	3,473	3,121	406	3,295	3,013	505	3,313	2,938	587	3,180	3,011	658	3,264	3,312	728	3,609	3,801
**Insulin therapy**	**898**	6,759	5,203	1,029	6,800	5,192	1,150	7,063	5,573	1,214	7,058	5,903	1,256	7,228	5,658	1,032	7,398	5,487
**Non diabetic patients**	**27,389**	**1,715**	**2,914**	**28,962**	**1,767**	**3,060**	**30,618**	**1,866**	**3,261**	**32,103**	**1,994**	**3,475**	**31,128**	**2,150**	**3,979**	**26,253**	**2,166**	4,102
**Patients with Declining Renal Function**																		
**Monotherapy**	**282**	4,148	4,258	**339**	4,267	4,426	**393**	4,396	4,811	**465**	4,897	6,427	**518**	4,149	4,637	**411**	4,226	5,369
**Double therapy**	**157**	3,773	3,144	**228**	3,603	3,628	**266**	3,667	3,257	**308**	3,892	4,004	**310**	3,891	4,623	**267**	4,159	5,268
**Triple therapy**	**27**	4,244	3,641	**41**	3,105	1,617	**45**	4,052	3,396	**69**	4,203	4,411	**71**	3,387	3,027	**78**	4,032	4,329
**Insulin therapy**	**136**	10,772	9,335	**188**	10,227	8,882	**240**	10,644	10,731	**294**	10,499	10,185	**359**	11,155	11,188	**267**	11,344	11,185

### Individual ambulatory care expenditures in 2010

In 2010, annual individual ambulatory care expenditures slightly increased from MT (€3,017 [95%CI: €3,015;€3,019]) to DT (€3,308 [€3,305;€3,311]), and from DT to TT (€3,609 [€3,599;€3,619]). Switching from OADs/GLP-1 analogs to insulin led to an increase in individual ambulatory care expenditures to €7,398 [€7,388;€7,408]. This substantial increase was also found in other years and is mainly attributable to nursing care (+1,000% vs. MT), medical devices (+347%), transportation (+200%) and pharmacy expenditures (+129%) (Table 5).

**Table 5:** Details of individual ambulatory care expenditures for patients with T2DM and normal renal function and for non-diabetic controls in 2010 (€)

	**Consultations visits**	**Physio-therapist**	**Nursing care**	**Biological diagnostic tests**	**Radiology/medical imaging**	**Medical devices**	**Transportation**	**Invalidity**	**Pharmacy**	**Others**	**Total ambulatory care costs**
**Monotherapy (mono)**: m (σ)	256 (190)	90 (339)	144 (853)	159 (175)	113 (191)	272 (861)	88 (449)	219 (1,451)	1,156 (1,786)	521 (1,528)	3,017 (3,829)
**Double therapy (bi)**: m (σ)	257 (156)	88 (372)	152(885)	155 (158)	107 (186)	294 (728)	98 (787)	240 (1,730)	1,411 (1,650)	506 (1,679)	3,308 (4,045)
**Cost ratio** vs mono	***1.045***	***0.909***	***1.392***	***1.070***	***0.940***	***1.237***	***1.113***	***0.976***	***1.236***	***0.885***	***1.102***
**Triple therapy (tri)**: m (σ)	252 (146)	55(280)	94 (646)	149 (129)	110 (188)	272 (410)	112 (707)	308 (1,857)	1,798 (1,509)	460 (1,337)	3,609(3,801)
**Cost ratio** vs bi	***1.044***	***0.728***	***0.675***	***0.977***	***1.038***	***1.059***	***1.022***	***1.026***	***1.287***	***0.926***	***1.101***
**Insulin therapy**: m (σ)	329 (208)	157 (499)	1,586 (2761)	198 (210)	140 (221)	1,216 (1,697)	264 (1,064)	444 (2,025)	2,646 (2,124)	420 (1,093)	7,398(5,487)
**Cost ratio** vs tri	***1.200***	***2.509***	***18.420***	***1.189***	***1.178***	***3.931***	***2.480***	***2.929***	***1.331***	***1.228***	***1.942***
**Non diabetic patients**	175 (184)	81 (324)	101 (977)	93 (176)	95 (170)	160 (805)	85 (582)	136 (1,131)	702 (2,142)	538 (1,799)	2,166 (4,102)
**Additional costs vs non diabetic patients: € (increase rate in %)**											
Monotherapy	80 (46.3%)	9 (11.1%)	43 (42.6%)	66 (71.0%)	18 (18.9%)	112 (70.0%)	3 (3.5%)	83 (61.0%)	454 (64.7%)	−17 (-3.2%)	852 (39.3%)
Double therapy	81 (46.9%)	7 (8.6%)	51 (50.5%)	62 (66.7%)	12 (12.6%)	134 (83.8%)	13 (15.3%)	104 (76.5%)	709 (101.0%)	−32 (-5.9%)	1,142 (52.7%)
Triple therapy	77 (44.0%)	−26 (-32.1%)	−7 (-6.9%)	56 (60.2%)	15 (15.8%)	112 (70.0%)	27 (31.8%)	172 (126.5%)	1,096 (156.1%)	−78 (-14.5%)	1,444 (66.7%)
Insulin therapy	154 (88.0%)	76 (93.8%)	1,485 (1,470.3%)	105 (112.9%)	45 (47.4%)	1,056 (660.0%)	179 (210.6%)	308 (226.5%)	1,944 (276.9%)	−118 (-21.9%)	5,234 (241.7%)

*Cost ratios from Generalized Linear Models (GLM), adjusted on year, socio demographic patient characteristics, long-term conditions, co-treatments.

The regression model showed that individual ambulatory expenditures increased by a ratio of 1.94 (p < 0.001) from TT to IT, with adjustment for socio-demographic characteristics and co-treatments. Nursing care costs increased by a ratio of 18.42 (p < 0.001), medical devices costs (including glucose monitoring device) by 3.93 (p < 0.001) and pharmacy costs by 1.33 (p < 0.001) (Table 5).

The drug costs increased with treatment escalation: from €1,113 (€3.04/day) in MT to €1,381 in DT (€3.78/day), to €1,701 (€4.66/day) in TT and to €2,615 (€7.16/day) in IT. Pharmacy is the first cost category accounting for 38% of ambulatory care expenditures in MT, 43% in DT, 50% in TT and 36% in IT (Tables 6 and 7).

**Table 6:** Impact of the treatment phase on the annual ambulatory healthcare expenditures attributable to diabetes for patients with T2DM with normal renal function (€) (Adjusted model on differences in expenditures between T2-DM patients and matched individuals without diabetes)

**Treatment phases (ref = monotherapy)**	**Pharmacy**	**Consultations**	**Medical devices**	**Nursing cares**	**Others**	**Total ambulatory costs**
**Double therapy**	238***	9***	26***	9	−31	224***
	(213;262)	(6;12)	(15;38)	(-5;23)	(-71;9)	(169;280)
**Triple therapy**	556***	16***	35**	−30*	−131***	411***
	(509;603)	(11;21)	(13;57)	(-56;-4)	(-208;-55)	(306;517)
**Insulin therapy**	1,224***	78***	863***	1,236***	1,268***	3,807***
	(1,190;1,259)	(74;82)	(847;880)	(1,217;1,256)	(1,212;1,325)	(3,730;3,885)

* 0.01 < p < 0.05; ** 0.001 < p < 0.01; *** p < 0.001.

**Table 7:** Details of medication costs by drug class and treatment phase (€)

	**Mono therapy**		**Double therapy**		**Triple therapy**		**Insulin therapy**		**Non diabetics**	
	**Mean**	**SD**	**Mean**	**SD**	**Mean**	**SD**	**Mean**	**SD**	**Mean**	**SD**
**A-Alimentary tract and metabolism**										
**Insulin**	€4	(€37)	€10	(€55)	€12	(€63)	€881	(€516)	€3	(€54)
**Oral antidiabetic agents**	€129	(€114)	€367	(€201)	€647	(€244)	€147	(€196)	-	-
**GLP-1 Analog**	€3	(€50)	€13	(€121)	€94	(€319)	€32	(€185)	-	-
**Other alimentary tract and metabolism products**	€75	(€136)	€64	(€111)	€64	(€116)	€121	(€174)	€55	(€125)
**B-Blood and blood forming organs**										
**Antithrombotic agents**	€61	(€154)	€69	(€172)	€61	(€170)	€127	(€291)	€34	(€120)
**Antihemorrhagics**	€0	(€1)	€1	(€68)	€0	(€0)	€0	(€3)	€0	(€55)
**Antianemic preparations**	€18	(€396)	€18	(€287)	€7	(€106)	€46	(€670)	€12	(€296)
**Blood substitutes and perfusion solutions**	€1	(€15)	€0	(€2)	€0	(€3)	€1	(€7)	€2	(€70)
**C- Cardiovascular system**										
**Cardiac therapy**	€24	(€87)	€20	(€72)	€15	(€52)	€43	(€120)	€14	(€57)
**Antihypertensive**	€10	(€49)	€13	(€55)	€15	(€61)	€19	(€65)	€4	(€27)
**Diuretics**	€14	(€52)	€12	(€37)	€11	(€66)	€24	(€77)	€6	(€34)
**Beta-blocking agents**	€33	(€62)	€33	(€60)	€33	(€58)	€43	(€76)	€15	(€40)
**Calcium channel blockers**	€31	(€65)	€33	(€66)	€32	(€67)	€40	(€72)	€14	(€43)
**Agents acting of the renin-angiotensin system**	€152	(€147)	€171	(€155)	€196	(€160)	€207	(€163)	€61	(€105)
**Lipid modifying agents – Statins**	€127	(€162)	€146	(€179)	€177	(€185)	€190	(€201)	€52	(€113)
**Lipid modifying agents - Other**	€19	(€76)	€19	(€81)	€20	(€89)	€24	(€109)	€10	(€53)
** Other cardiovascular system drugs**	€4	(€23)	€4	(€24)	€5	(€30)	€8	(€36)	€2	(€17)
**D-Dermatologicals**	€10	(€40)	€11	(€43)	€13	(€61)	€18	(€61)	€7	(€32)
**G-Genito urinary system and sex hormone**	€15	(€65)	€13	(€58)	€12	(€51)	€18	(€72)	€14	(€52)
**H-Systemic hormonal preparations, excl. sex hormones and insulins**	-	-	-	-	-	-	-	-	€8	(€192)
**J-Antiinfectives for systemic use**	€39	(€419)	€36	(€418)	€19	(€39)	€69	(€416)	€38	(€492)
**L-Antineoplastic and immunomodulating agents**	€70	(€881)	€82	(€921)	€27	(€233)	€141	(€1371)	€78	(€906)
**M-Musculo-skeletal system**	€27	(€88)	€25	(€63)	€27	(€121)	€24	(€56)	€28	(€98)
**N-Nervous system**	€0	(€0)	€0	(€0)	€0	(€0)	€0	(€0)	€0	(€0)
**Psychotropics**	€50	(€241)	€42	(€183)	€44	(€145)	€82	(€246)	€33	(€146)
**Other nervous system product**	€91	(€255)	€92	(€269)	€92	(€247)	€181	(€430)	€74	(€293)
**P-Antiparasitic products, insecticides and repellents**	€0	(€5)	€0	(€3)	€1	(€6)	€1	(€7)	€0	(€0)
**R-Respiratory system**	€58	(€223)	€46	(€173)	€46	(€179)	€86	(€295)	€43	(€188)
**S-Sensory organs**	€38	(€279)	€32	(€230)	€24	(€118)	€33	(€193)	€29	(€275)
**V-Various**	€9	(€139)	€8	(€72)	€5	(€17)	€8	(€28)	€9	(€255)
**Total**	€1113	(€1,411)	€1,381	(€1,435)	€1,701	(€868)	€2,615	(€2,089)	€644	(---)

**Figure 3:**
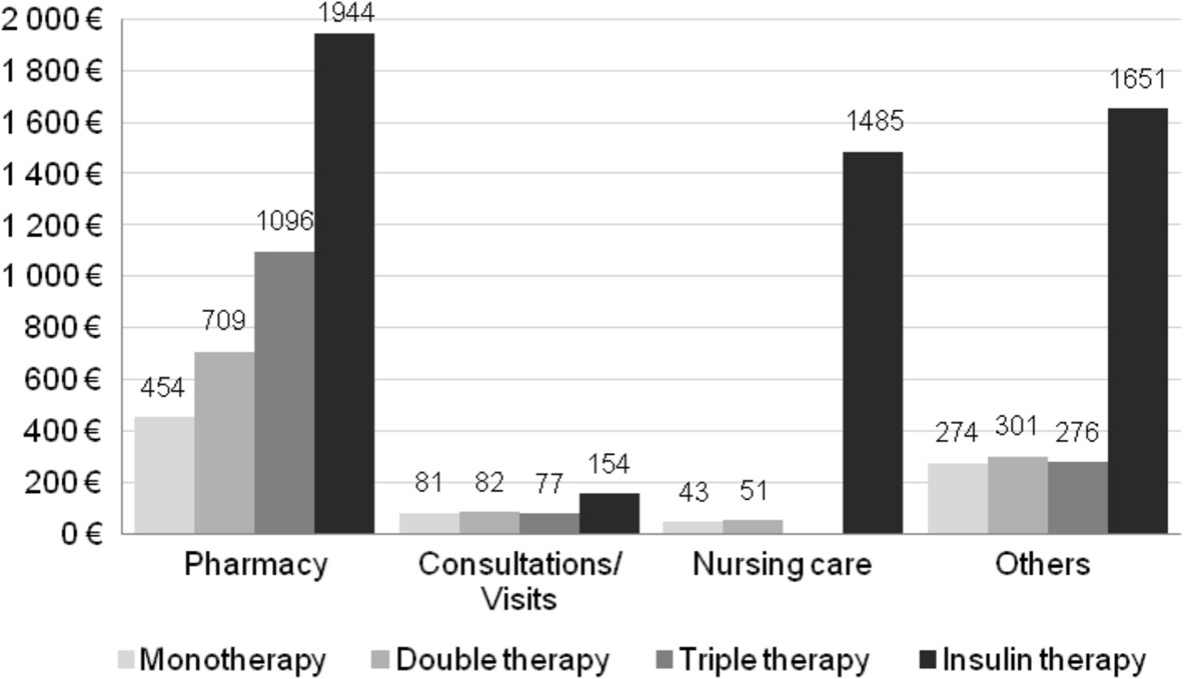
Additional individual direct drug costs attributable to T2DM by treatment phase and resource category in 2010.*% calculated by treatment line; ex: additionnal drug costs in monotherapy = 28% antidiabetics, 51% cardiovascular system, 7% nervous system 7% blood and blood forming organs, 7% others.

Average costs of antidiabetic drugs in 2010 (insulin/OADs/GLP-1 analogs) were €136 (€0.37/day) in MT, €390 (€1.07/day) in DT, €753 (€2.06/day) in TT and €1,060 (€179 for OADs and €881 for insulin, €2.90/day) for patients with insulin (Table 7). The cost of insulin alone does not explain the substantial cost increase when moving to this final treatment phase (Figure 3; Table 6).

**Figure 4:**
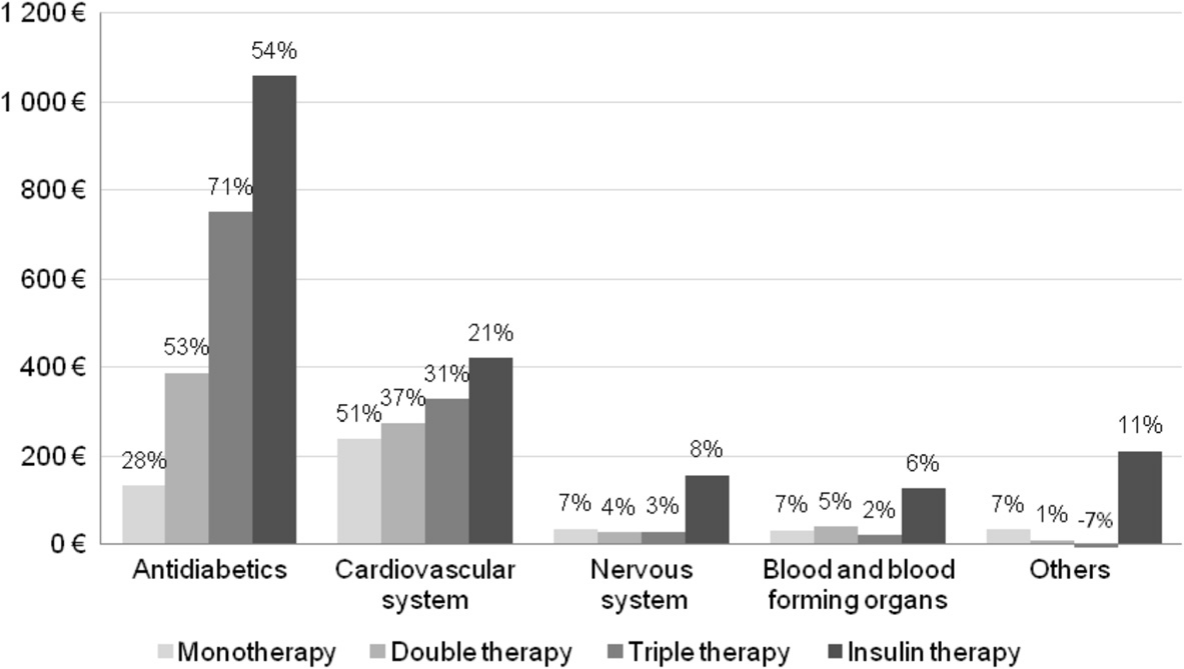
Additional individual ambulatory care expenditures attributable to T2 DM by treatment phase and resource category in 2010*.*% calculated by treatment line; ex: additional costs in monotherapy = 53% pharmacy + 9% consultations + 5% nursing care + 32% others.

DM-attributable expenditures mainly consist of drug costs for patients without insulin (€1,096 for TT). In addition, significantly higher DM-attributable costs related to nursing care (€1,485) and medical devices (€1,056) are incurred for patients with insulin (Figure 4). DM-attributable drug costs are largely related to antidiabetic drugs (from 28% in MT to 71% in TT; 54% in IT), and also to cardiovascular system drugs (21% in IT to 51% in MT) and nervous system drugs (from 3% in TT in to 8% in IT) as shown in Figure 3.

The regression analysis shows an increase of €224/patient in the ambulatory expenditures attributable to T2DM between MT and DT, i.e + 26% (see Table 7). This difference reached €411 between MT and TT (+48%) but the largest increase was observed during the transition to insulin, equal to €3807 (+447%) and €3396 (+269%) compared to MT and TT respectively. This analysis also showed substantial increase rates associated with the transition to insulin, for other resource categories, especially nursing care.

### Patients with declining renal function

Individual ambulatory care expenditures for patients with DRF exceeded those for patients with NRF by 12% (TT) to 53% (IT), with estimated totals of €4,226 [€4,200;€4,252] for MT in 2010, €4,159 [€4,120;€4,198] for DT, €4,032 [€3,923;€4,141] for TT and €11,344 [€11,262;€11,426] for IT (Table 8). The same cost pattern was found for previous years.

**Table 8:** Individual ambulatory care expenditures for patients with type T2DM and declining renal function in 2010 (€)

		**Pharmacy**	**Consultation (GP/specialist)**	**Nursing care**	**Total ambulatory care costs**
Monotherapy (N = 411)	m (σ)	1,798 (2,433)	307 (252)	143 (683)	4,226 (5,369)
	Increase rate vs NRF	56%	20%	0%	40%
Double therapy (N = 267)	m (σ)	1,893 (3,908)	301 (171)	210 (963)	4,159 (5,268)
	Cost ratio vs mono (%)	1.094*	0.978	0.736	0.959
	Increase rate vs NRF	34%	21%	38%	26%
Triple therapy (N = 78)	m (σ)	2,141 (3,056)	305 (152)	99 (504)	4,032 (4,329)
	Cost ratio vs mono (%)	1.262 ***	1.029	0.903	1.031
	Increase rate vs NRF	19%	17%	5%	12%
Insulin therapy (N = 267)	m (σ)	3,889 (5,363)	422 (340)	2,161 (3,638)	11,344 (11,185)
	Cost ratio vs mono (%)	2.020 ***	1.230 ***	10.236 ***	2.223***
	Increase rate vs NRF	47%	28%	36%	53%

* 0.01 < p < 0.05; ** 0.001 < p < 0.01; *** p < 0.001.

## Discussion

The results of this study show that individual ambulatory care expenditures for patients with T2DM increased moderately and progressively from 2005 to 2010. Increase rates were lower for patients with T2DM in a stable treatment stage (< 15%), than for controls without diabetes (26%) with an average annual rate (from 0.9% for TT to 2.8% for DT) close to the inflation rate (from 1.5% to 2.8%) over the same period (except 2009). The introduction of new classes of antidiabetic drugs in 2008-2009 (DPP4 and GLP-1 analogs) did not appear to have any substantial impact on overall health care expenditures for DM patients over the considered period. In addition, individual yearly ambulatory care expenditures increased with treatment escalation. Insulin therapy was associated with substantial cost increase compared to earlier stages of treatment, related to pharmacy but also nursing care and medical devices utilisation. Additional drug costs attributable to diabetes are not only related to antidiabetic drugs and insulin but also to other drugs, mainly cardiovascular system and psychotropic drugs (antidepressants and analgesics). Although adjustments for patient socio-demographics and co-treatments were used, it cannot be ruled out that a residual part of the estimated difference in expenditures between insulin-treated patients and other DM patients is attributable to worsening health status. However, a large part of those additional expenditures appears to be directly related to administration of insulin, as suggested by high rates of increase in costs of nursing care and medical devices.

In addition, individual yearly expenditures for DM patients with DRF were showed to exceed expenditures for other DM patients by +12% (TT) to +53% (IT), again with substantial nursing care and medical device costs for patients treated with insulin.

The estimated expenditures reported here are consistent with results of previous studies on the costs of diabetes in France. Ambulatory care expenditures were approximately €3,400 in 2007, among patients with or without insulin, compared to €3,300 (excluding hospitalisation) in ENTRED. The pharmacy expenditures estimates (€1,156, €1,411 and €1,798 in MT, DT and TT) are also comparable to those from the ENTRED study (around €1,400) [4]. A review on costs of diabetes in France suggested that costs in patients with insulin are about twice as much as in patients treated with oral antidiabetics, which is also consistent with our findings [7]. However, the cost difference between patients with and without insulin was thought to be largely attributable to complications [8]. In addition, the importance of nursing care costs in patients with insulin is corroborated by a recent French publication focusing on the costs associated with insulin therapy [9]: nursing care (€25.8/week) was the most important contributor to the costs of insulin-therapy (€45.4/week).

Real-world healthcare expenditures were measured in this study by analyzing health insurance claims data. The strengths of the EGB database, relative to other health insurance databases in Western countries, include its size, its representativity, the absence of selection according to clinical or socioeconomic criteria, and the fact that most persons are continuously enrolled. However, this study has limitations related to the utilisation of administrative data, rather than clinical data. Thus, the identification of patients with DRF was based on very restrictive assumptions. We estimated the proportion of patients with DRF at 10%, whereas higher estimates have been reported [10,11]. This implies that the groups of patients with NRF probably included a few patients with DRF.

The EGB database contains no other socio-demographic data than age, sex, date of death, residence department and the affiliation in the CMU. Education level, income level or occupational category of beneficiaries, for which the influence on health care consumption has been demonstrated [12,13], are absent from the EGB. This information should be collected through specific surveys.

The description of the results over time does not consider all treated DM patients but only focuses on the subpopulation of DM patients that are stable in their treatment. Patients not stable or switching from a treatment to another within the year were excluded from the analysis. This facilitated the comparison of expenditures between treatment stages but also introduced a selection bias that might affect the description of expenditures for the overall population. In particular, expenditures of switching between different treatment stages were not captured. The reported estimations are therefore conservative, reflecting the most stable and compliant patients.

Some patients in a stable treatment phase were not identified in 2010, as suggested by patient numbers by year. The selection method was based on restrictive criteria and the absence of data after December 2010 led to exclude several patients, classified as lost to follow-up.

## Conclusions

In conclusion, individual ambulatory care expenditures are substantially higher among patients with T2DM in a stable treatment phase, but have grown at a slower rate than in controls without diabetes with same age and gender over recent years up to 2010. Pharmacy is the first category of ambulatory care expenditures associated with diabetes; this is not only due to costs of antidiabetic drugs, but also to treatment of comorbidities. This study also highlighted two key determinants of ambulatory care expenditures among patients with T2DM: treatment stage and renal function status. Individual ambulatory care expenditures increase with treatment escalation, and most particularly transition to insulin therapy, because of pharmacy costs, nursing care and medical devices utilisation. Expenditures for patients with DRF are higher than for patients without DRF, at all treatment stages, and particularly for insulin users. These findings provide an economic argument for maintaining the diabetic patients under oral treatments as long as they can be controlled by oral treatment. Finally, this study suggests that the introduction of new oral antidiabetic treatments had little impact on the total ambulatory care expenditure for patients with T2DM.

## Abbreviations

ACE-I: Angiotensin converting enzyme inhibitors; ALD: Affections longues durées (long term conditions); A2RA: Angiotensin II receptor antagonists; CMU: Couverture mutuelle universelle (“basic” universal health cover); CRD: Chronic renal disease; DPP4: Dipeptidyl peptidase-4 inhibitors; DRF: Declining renal function; DT: Double therapy; EGB: Echantillon generaliste de beneficiaires (general sample of beneficiaries); ENTRED: Echantillon national témoin représentatif des personnes diabétiques; GLP-1: Glucagon-like peptide-1; HAS: haute autorité de Santé (the French national authority for health); IT: Insulin therapy; OADs: Oral antiDiabetics; MT: Monotherapy; T2DM: Type 2 diabetes mellitus; TT: Triple therapy.

## Competing interests

Duality of interest: The Department of Pharmacology at Bordeaux University does studies of drug effectiveness and risks in real-life use with various study designs. Studies are done at the request of the regulatory authorities, or for their attention. Most of these studies are funded by pharmaceutical companies, including most manufacturers of insulin and other glucose-lowering products.

## Authors’ contributions

FG participated in the design of the study, supervised the analysis, interpreted the data and drafted the manuscript. EC participated in the design of the study, performed the data management and the statistical analysis and contribute in the interpretation of the data. SA participated in the design of the study, contributed in the interpretation of data and to drafting the manuscript. RG participated in the design of the study, contributed in the interpretation of data and revised the manuscript critically for important intellectual content. NM participated in the conception of the study, revised the manuscript critically for important intellectual content and gave final approval of the version to be published. MT participated in the conception of the study, revised the manuscript critically for important intellectual content and gave final approval of the version to be published. All authors read and approved the final manuscript.

## Pre-publication history

The pre-publication history for this paper can be accessed here:

http://www.biomedcentral.com/1472-6823/13/15/prepub
